# A rare case of pregnancy-associated thrombotic thrombocytopenic purpura

**DOI:** 10.1093/omcr/omag099

**Published:** 2026-06-15

**Authors:** Ziad W Elmezayen, Enas Samara, Alaa Zayed, Tujan Hamed

**Affiliations:** Faculty of Medicine, Kafr Elsheikh University, Kafr Elsheikh, Egypt; Department of Medicine, Faculty of Medicine and Health Sciences, An-Najah National University, Nablus, Palestine; Department of Medicine, Faculty of Medicine and Health Sciences, An-Najah National University, Nablus, Palestine; Department of Medicine, Faculty of Medicine and Health Sciences, An-Najah National University, Nablus, Palestine

**Keywords:** thrombotic thrombocytopenic purpura, TTP, pregnancy-associated TTP, ADAMTS13, thrombotic microangiopathy, plasma exchange, HELLP syndrome, third trimester, maternal outcomes

## Abstract

Thrombotic Thrombocytopenic Purpura (TTP) is a rare but potentially life-threatening thrombotic microangiopathy that presents significant diagnostic and therapeutic difficulties during pregnancy due to overlap with conditions like HELLP syndrome and preeclampsia. We present the case of a previously healthy 28-year-old woman at 34 weeks of gestation presenting with fatigue, headache, and petechiae. Laboratory tests indicated anemia, thrombocytopenia, increased lactate dehydrogenase levels, and the presence of schistocytes in the peripheral smear. Low ADAMTS13 activity (<10%) confirmed TTP. The patient showed significant improvement after receiving corticosteroids and undergoing daily plasma exchange. For maternal stability, an elective caesarean section was performed at 36 weeks, resulting in good maternal and neonatal outcomes. Early detection requires the timely diagnosis of unexplained thrombocytopenia, hemolysis, and neurologic symptoms in the absence of hypertension or abnormal coagulation, which should prompt immediate ADAMTS13 testing. Diagnosis and treatment were guided by multidisciplinary management and ADAMTS13 testing, resulting in complete remission without relapsing at the six-week follow-up.

## Introduction

Thrombotic Thrombocytopenic Purpura (TTP) is a rare, life-threatening thrombotic microangiopathy marked by thrombocytopenia and microangiopathic hemolytic anemia, with cognitive deficits, renal impairment, and fever [1]. It is caused by severe ADAMTS13 enzyme deficiency, either congenital, more frequently, acquired through inhibitory autoantibodies, leading to the accumulation of ultra-large von Willebrand factor (vWF) multimers and widespread microvascular thrombosis [2].

TTP is triggered by pregnancy, especially during the third trimester and postpartum period. Increased vWF and decreased ADAMTS13 activity, along with physiological changes during pregnancy, can exacerbate a prothrombotic condition [2]. The incidence of pregnancy-associated TTP is 1 in 25 000 pregnancies. Its symptoms can postpone necessary treatment since they overlap with those of more prevalent maternal problems, including preeclampsia and HELLP syndrome [3]. Delayed diagnosis can be fatal, but early plasma exchange has reduced maternal mortality from 90% to much lower rates today [4].

Effective management requires a multidisciplinary team, particularly in atypical early-onset cases that complicate diagnosis and treatment [4]. The purpose of this case report is to highlight the critical clinical signs of early TTP diagnosis in pregnancy, as well as multidisciplinary care for better outcomes.

## Case presentation

A 28-year-old gravida 2 para 1 woman, at 34 weeks of gestation, presented with severe fatigue, headache, and the appearance of a petechial rash over the lower extremities of one week’s duration. She denied any visual disturbances or abdominal pain. Her prior pregnancy was uncomplicated, and her current pregnancy had been uneventful until the current presentation. Family history was non-contributory for hematologic or thrombotic disorders.

On examination, she appeared fatigued but was alert and oriented. Her vital signs were stable. Petechiae were noted over the lower extremities, and mild splenomegaly was palpated on abdominal examination. There were no jaundice, mucosal bleeding, or other skin lesions observed. Laboratory investigations at admission are shown in Table 1, revealing anemia, thrombocytopenia, elevated lactate dehydrogenase, indirect hyperbilirubinemia, and normal liver and renal function. Peripheral blood smear showed schistocytes, consistent with microangiopathic hemolytic anemia. Coagulation parameters and urinalysis. The differential diagnosis included HELLP syndrome and preeclampsia; however, the absence of hypertension, proteinuria, and elevated liver enzymes made these less likely. ADAMTS13 activity was found to be < 10%, confirming the diagnosis of TTP. Due to restricted local availability, no testing for ADAMTS13 inhibitors (e.g. Bethesda assay) or genetic analysis for ADAMTS13 mutations was undertaken. A diagnosis of pregnancy-associated with TTP was established based on the clinical and laboratory findings.

**Table 1:** Laboratory testing.

Parameter	Result	Reference Range
Hemoglobin	8.5 g/dl	12–16 g/dl
Platelet Count	45 000/μl	150–450 × 10^3^/μl
Lactate dehydrogenase (LDH)	450 U/l	140–280 U/l
Indirect bilirubin	1.5 mg/dl	0.2–1.2 mg/dl
Serum Creatinine	0.9 mg/dl	0.6–1.1 mg/dl

The patient was started on daily plasmapheresis, completing a total of 10 sessions over two weeks. She also received intravenous methylprednisolone at a dose of 1 mg/kg/day for three days, followed by a tapering course of oral corticosteroids. Red blood cell transfusions were used to treat symptomatic anemia. Low-dose aspirin was administered despite a platelet count of 45 000/μl because the patient had no active bleeding or mucosal hemorrhage, and the decision was made jointly by hematology and obstetrics with daily platelet monitoring and continued until platelet normalization. Fetal monitoring was maintained throughout the treatment course. An elective caesarean section was performed at 36 weeks of gestation for maternal stability. The newborn was admitted to the neonatal intensive care unit for observation, but no complications occurred. The infant had a normal birth weight and clinical examination. Following delivery and completion of therapy, the patient demonstrated significant clinical improvement. Her symptoms were resolved, and follow-up laboratory studies showed normalization of hemoglobin, platelet count, and hemolysis markers. She was discharged on postpartum day 3 in stable condition and remained asymptomatic at her six-week follow-up, with no recurrence of TTP. The following table summarizes the key clinical events from symptom onset through postpartum recovery (Table 2).

**Table 2:** Clinical timeline.

Day	Clinical Event
Day 1	Symptoms began at 33 weeks’ gestation
Day 7	Admission began at 34 weeks’ gestation, labs drawn; TTP suspected ADAMTS13 activity < 10% confirmed; plasmapheresis initiated
Day 7–21	Daily plasmapheresis (10 sessions total); corticosteroids started
Day 21	Elective caesarean section at 36 weeks Neonate admitted to NICU for observation (no complications)
Day 22–24	Continued postpartum monitoring; clinical improvement
Day 24	Discharged home on postpartum day 3; stable labs and no symptoms
6-week FU	Follow-up: asymptomatic, normal labs, no recurrence

## Discussion

TTP results from a severe deficiency of the ADAMTS13 enzyme, which normally regulates the size and activity of von Willebrand factor (vWF) multimers. When ADAMTS13 activity falls below 10%, excess vWF promotes platelet aggregation in small vessels, leading to thrombotic microangiopathy and multiorgan ischemia. Physiological stressors such as infection, inflammation, or pregnancy often trigger clinical flare-ups by increasing endothelial activation and vWF release.

TTP’s differential diagnosis includes pregnancy-related Thrombotic Microangiopathies such as HELLP syndrome and preeclampsia, hemolytic uremic syndrome, secondary causes like drugs, malignancy, autoimmune disease, or transplantation, as well as DIC, severe B12 deficiency, autoimmune cytopenias, hematologic malignancies, and malignant hypertension. Clinical context, laboratory findings, and lower ADAMTS13 activity (<10%) are key factors that differentiate most other conditions from TTP.

This case was challenging due to the clinical overlap between TTP and HELLP syndrome; both conditions cause thrombocytopenia and hemolysis in the third trimester. However, the absence of hypertension and proteinuria, along with the presence of neurological symptoms and a higher likelihood of TTP, helped differentiate the two. The patient had normal blood pressure (120/80 mmHg), no proteinuria, and normal liver enzymes. The presence of neurological symptoms and petechiae, together with ADAMTS13 deficiency (<10%), confirmed TTP over HELLP. Early testing and multidisciplinary coordination were essential in balancing treatment and delivery timing at 33 weeks.

In our case, the early suspicion of TTP was crucial for achieving a favorable outcome. Early detection of TTP during pregnancy relies on identifying specific clinical and laboratory features that distinguish it from other thrombotic microangiopathies. The patient experienced fatigue, headache, petechiae, anemia, and thrombocytopenia, which indicated thrombotic microangiopathy. Blood smear revealed schistocytes. Labs showed elevated LDH, indirect hyperbilirubinemia, and a low platelet count. ADAMTS13 activity was less than 10%, confirming TTP.

Treatment includes plasma exchange and corticosteroids. Congenital TTP requires plasma infusions. Refractory cases may need immunosuppressants such as azathioprine, ciclosporin, and rituximab. Caplacizumab is not recommended due to fetal concerns. Low-dose aspirin and low-molecular-weight heparin can be used if platelet counts exceed 50 × 10^9^/l [5].

However, in our patient, low-dose aspirin was initiated at a platelet count of 45 000/μl in the absence of bleeding manifestations, with close daily monitoring—an individualized decision that may not be generalizable. Monitoring involves regular blood counts, ADAMTS13 levels, and fetal assessments to keep ADAMTS13 above 20%–25%. Although vaginal delivery is preferred in hemodynamically stable patients with normal platelet counts, caesarean section was chosen in this case to avoid the unpredictable hemodynamic and platelet demands of labor, given the recent resolution of acute TTP and the risk of peripartum relapse. Importantly, delivery does not treat TTP. Plasma treatment and vigilant monitoring are part of postpartum care. Pre-pregnancy rituximab is used to prevent recurrence in women with low ADAMTS13 levels, and subsequent pregnancies are closely monitored [2]. Plasma exchange was used to remove autoantibodies and restore ADAMTS13 activity, while corticosteroids suppressed the immunological response. Aspirin reduces the risk of clotting, and anemia is treated with red blood cell transfusions. Platelet transfusions are avoided. Cesarean delivery is performed at 36 weeks.

Because no ADAMTS13 inhibitor testing nor genetic analysis was performed, we cannot distinguish between acquired autoimmune TTP (most likely given the acute pregnancy trigger) versus congenital TTP (Upshaw–Schülman syndrome). This distinction has major implications for future pregnancies: if acquired, repeat pregnancy carries a 20%–30% relapse risk, and prophylactic plasma exchange with corticosteroid taper should be considered; if congenital, she would require prophylactic plasma infusions every 2–3 weeks during any future pregnancy. We have advised the patient to undergo comprehensive ADAMTS13 testing (activity, inhibitor, and genetic analysis) prior to her next pregnancy and to be referred to a specialized thrombotic microangiopathy center for preconception counseling.

Long-term follow-up is important due to relapse and associations with autoimmune disorders such as lupus and Sjögren’s. Persistent ADAMTS13 deficiency during remission predicts relapse, and preemptive treatment with rituximab may be considered to lower the probability of recurrence. Follow-up should also assess for complications such as neurocognitive deficits, arterial hypertension, and major depression [6].

This case provides further evidence that, for better outcomes for both the mother and the fetus, early detection and rapid plasma exchange initiation, guided by ADAMTS13 activity and multidisciplinary therapy, are essential. Our results are consistent with those of Nikolaou et al. [7], showing that even in severe, late-pregnancy cases, full remission can be attained with prompt diagnosis and focused treatment.

## Conclusion

This case highlights the importance of considering TTP in pregnant women with thrombocytopenia and hemolytic anemia. It emphasizes the necessity of early ADAMTS13 testing, multidisciplinary care, and prompt therapy for better maternal and fetal outcomes.
